# Reframing Buprenorphine as a Pharmacologic Modifier of Opioid-Induced Respiratory Depression in the Fentanyl Era

**DOI:** 10.3390/ph19050799

**Published:** 2026-05-20

**Authors:** Anees Bahji, Imran Ghauri, Nickie Mathew, Nathaniel Day, Robert Tanguay

**Affiliations:** 1 Department of Psychiatry, Cumming School of Medicine, University of Calgary, Calgary, AB T2N 4N1, Canada; 2 Department of Community Health Sciences, Cumming School of Medicine, University of Calgary, Calgary, AB T2N 1N4, Canada; 3 Mathison Centre for Mental Health Research & Education, Hotchkiss Brain Institute, University of Calgary, Calgary, AB T2N4Z6, Canada; 4 Canadian Centre of Recovery Excellence, Edmonton, AB T6E 5A6, Canada; 5 Recovery Alberta, Edmonton, AB T5J 3E4, Canada; 6 Department of Psychiatry, University of British Columbia, Vancouver, BC V6T 1Z4, Canada; 7 BC Mental Health and Substance Use Services, Provincial Health Services Authority, Vancouver, BC V5C 6E3, Canada; 8 Department of Family Medicine, Faculty of Medicine & Dentistry, University of Alberta, Edmonton, AB T6G 2T4, Canada; 9 Department of Surgery, Cumming School of Medicine, University of Calgary, Calgary, AB T2N 1N4, Canada

**Keywords:** buprenorphine, opioid-induced respiratory depression, fentanyl, overdose prevention, opioid agonist therapy, harm reduction

## Abstract

The overdose crisis in North America is increasingly driven by illicitly manufactured fentanyl and other high-potency synthetic opioids, which are associated with severe and unpredictable opioid-induced respiratory depression (OIRD). Current pharmacologic strategies to prevent fatal overdose have largely emphasized downstream rescue through opioid antagonism (e.g., naloxone), leaving limited attention to upstream pharmacologic modification of respiratory risk. In this Opinion article, we argue that buprenorphine should be reframed not only as a treatment for opioid use disorder (OUD), but also as a pharmacologic modifier of OIRD risk in fentanyl-dominant drug markets. Drawing on its partial μ opioid receptor agonism, ceiling effect on respiratory depression, and exceptionally high receptor affinity, we describe how buprenorphine can displace full agonists while limiting respiratory suppression. We further situate this pharmacology within emerging population-level observations from North American fentanyl contexts, suggesting reduced overdose mortality among individuals receiving opioid agonist therapy, particularly buprenorphine. In fentanyl-dominant drug markets, reframing buprenorphine as a modifier of respiratory risk has direct implications for clinical messaging about overdose protection, medication selection for individuals with ongoing illicit opioid use, and policy approaches aimed at reducing opioid-related mortality.

## 1. Introduction

Opioid-induced respiratory depression (OIRD) remains the proximate cause of death in most fatal opioid overdoses [1,2]. In the current era, this risk is amplified by the dominance of illicitly manufactured fentanyl and fentanyl analogues, which exhibit high potency, rapid central nervous system penetration, and substantial variability in dose and purity [3]. These features undermine traditional assumptions about opioid tolerance and erode the partial protection historically associated with stable patterns of opioid use.

Pharmacologic responses to OIRD have largely focused on downstream rescue, most notably through widespread naloxone distribution [4,5]. While naloxone has unquestionably saved countless lives, its reactive role leaves a substantial gap in prevention [6]. Compared with downstream rescue strategies, less attention has been paid to upstream pharmacologic approaches that may reduce the likelihood or severity of respiratory depression before it occurs [7,8].

Buprenorphine, a partial μ-opioid receptor agonist widely used in the treatment of opioid use disorder (OUD), is typically framed in terms of addiction treatment, retention in care, and craving suppression [9]. This framing, while valid, is incomplete. In fentanyl-dominant drug markets, buprenorphine’s pharmacologic properties—partial agonism, high receptor affinity, and a ceiling effect on respiratory depression—suggest a broader role in modifying respiratory risk, including among individuals who continue to use illicit opioids. Prior work has characterized buprenorphine’s respiratory effects in experimental and clinical settings and established its effectiveness as an OUD treatment. However, its potential role as a pharmacologic modifier of OIRD risk in real-world fentanyl environments has not been explicitly articulated. Integrating pharmacologic principles with contemporary overdose epidemiology supports a reframing of buprenorphine as part of a broader strategy for overdose prevention. The scope of this Opinion is grounded primarily in North American fentanyl-dominant drug markets, where illicitly manufactured fentanyl has become the principal driver of overdose mortality. Although the epidemiologic context varies across regions, the pharmacologic principles discussed here may also be relevant in other settings characterized by exposure to high-potency synthetic opioids.

Accordingly, this opinion advances a mechanistically grounded reframing of buprenorphine as a pharmacologic modifier of OIRD risk, synthesizing experimental, clinical, and population-level evidence to clarify its potential role in reducing overdose lethality and to inform clinical decision-making, harm reduction strategies, and policy development in fentanyl-dominant drug environments.

## 2. Opioid-Induced Respiratory Depression in the Fentanyl Era

Opioid-induced respiratory depression arises primarily through μ-opioid receptor-mediated suppression of brainstem respiratory centers, resulting in reduced respiratory rate, tidal volume, and responsiveness to hypercapnia and hypoxia [4,10,11]. High-potency synthetic opioids such as fentanyl further amplify these effects through rapid receptor occupancy and steep dose–response relationships [12,13].

Fentanyl’s pharmacokinetic and pharmacodynamic properties present several challenges. First, its high lipid solubility allows rapid penetration of the central nervous system, narrowing the margin between intoxication and respiratory failure [14,15,16]. Second, its high potency and frequent co-occurrence with other sedatives, including benzodiazepines, alcohol, xylazine, and medetomidine, increase the risk of synergistic respiratory suppression [17,18,19]. Third, the unpredictability of illicit supply hampers user titration strategies, even among individuals with significant opioid tolerance [20,21]. In this context, OIRD risk is not solely a function of the quantity of opioid exposure, but also of receptor dynamics and competitive agonism—an observation central to understanding buprenorphine’s potential role [22].

At the molecular level, μ-opioid receptor activation inhibits adenylate cyclase activity, reduces neuronal excitability by activating potassium channels, and suppresses neurotransmitter release by inhibiting calcium channels. Within the pre-Bötzinger complex and related brainstem respiratory networks, these effects blunt respiratory rhythm generation and impair ventilatory responsiveness. Together, these mechanisms help explain why fentanyl and other high-potency synthetic opioids can produce rapid, severe, and sometimes refractory respiratory toxicity.

## 3. Buprenorphine Pharmacology Relevant to Respiratory Risk

Buprenorphine possesses several pharmacologic properties that are directly relevant to opioid-induced respiratory depression (OIRD) risk [23,24,25]. These properties distinguish it from full μ-opioid receptor agonists and underpin its potential role as a modifier of respiratory toxicity in fentanyl-dominant drug environments. Clinically, buprenorphine is commonly prescribed in sublingual daily doses ranging from 2 to 24 mg for opioid use disorder, with higher receptor occupancy generally achieved at doses of 16 mg or greater [26]. Its terminal half-life after sublingual administration is typically reported to be approximately 24 to 42 h, supporting sustained receptor occupancy across dosing intervals.

Partial μ-Opioid Receptor Agonism and Ceiling Effect

As a partial agonist, buprenorphine produces submaximal intrinsic activity at the μ-opioid receptor [26]. A key consequence is a ceiling effect on respiratory depression: beyond a certain level of receptor occupancy, additional dosing does not result in proportional increases in respiratory suppression [27,28,29]. Experimental human studies have shown that buprenorphine-induced respiratory depression plateaus over the therapeutic dose range, in contrast to full agonists such as fentanyl, heroin, and methadone, which exhibit more linear or supralinear dose–response relationships with progressively greater respiratory depression at higher doses [7,30]. This pharmacodynamic distinction is central to buprenorphine’s improved safety profile.

High Receptor Affinity and Functional Antagonism

Buprenorphine’s exceptionally high affinity for the μ-opioid receptor—reported in the subnanomolar range in vitro and exceeding that of many full agonists, including fentanyl—allows it to displace other opioids and resist displacement once bound [31,32,33]. This high-affinity binding confers functional antagonism in the presence of full agonists. In fentanyl-exposed individuals, buprenorphine occupancy may limit the ability of fentanyl to achieve maximal receptor activation, thereby attenuating its respiratory depressant effects [34,35,36,37]. Importantly, this effect does not require complete abstinence from illicit opioids, making it relevant in real-world settings characterized by intermittent or ongoing exposure.

Pharmacokinetic Stability

Buprenorphine’s long receptor binding duration and relatively stable plasma concentrations contribute to sustained receptor occupancy and reduced fluctuation in opioid effect [9,29,33,37,38]. Sublingual bioavailability is limited and variable, commonly reported at roughly 30%, but once absorbed, the medication’s prolonged elimination and receptor persistence may mitigate periods of heightened vulnerability associated with short-acting, high-potency opioids. Taken together, these pharmacodynamic and pharmacokinetic properties suggest that buprenorphine is not simply less potent or less dangerous than full agonists, but qualitatively different in its interaction with respiratory control systems. This distinction supports its conceptualization as a pharmacologic modifier of respiratory risk rather than solely a substitution therapy.

## 4. Emerging Population-Level Observations

Although this article does not present new empirical analyses, a growing body of observational data from North America supports the clinical relevance of buprenorphine’s pharmacology in fentanyl-dominant settings [39]. Multiple population-based studies have demonstrated substantially reduced all-cause and overdose-specific mortality among individuals receiving opioid agonist therapy compared with those not in treatment [40]. In several jurisdictions, buprenorphine appears to confer mortality protection comparable to, and in some analyses greater than, methadone [25,40,41,42,43,44,45,46]. These observations are often interpreted primarily as evidence of treatment engagement, reduced illicit opioid use, or improved social stability [47,48,49,50]. While these mechanisms are undoubtedly important, they likely coexist with a direct pharmacologic effect on respiratory risk [51]. In fentanyl-contaminated markets, where intermittent exposure may continue despite treatment, the presence of a high-affinity partial agonist may blunt the lethality of otherwise fatal exposures [34,35,50,52]. In many markets, illicit opioids are contaminated with benzodiazepines. Evidence is emerging that buprenorphine can mitigate the risk of mixed benzodiazepine and opioid overdose [53]. Recognizing this mechanism does not negate the importance of social and behavioural pathways; rather, it enriches the causal framework for understanding observed mortality reductions [54,55].

## 5. Complementarity with Naloxone and Harm Reduction

Reframing buprenorphine as a modifier of OIRD risk should not be misconstrued as positioning it as an alternative to naloxone or other harm reduction interventions [56,57,58,59]. Rather, these approaches operate at different points along the overdose trajectory. The evidence supporting this perspective is derived largely from observational studies in North American populations with opioid use disorder, many of whom are characterized by polysubstance use, variable treatment engagement, and broader social and structural vulnerability.

Naloxone and nalmefene remain indispensable for the reversal of established respiratory depression through competitive μ-opioid receptor antagonism [60,61,62,63]. These agents act downstream, restoring respiratory function after significant opioid-induced suppression has already occurred. In contrast, buprenorphine exerts a distinct pharmacologic role. Through high-affinity partial agonism, it may reduce the likelihood, severity, or rate of onset of respiratory depression before it becomes clinically critical.

These mechanisms are therefore complementary rather than competing, supporting a layered approach to overdose prevention [64,65]. Naloxone intervenes after respiratory compromise has developed [63,66], whereas buprenorphine may shift baseline risk by attenuating receptor-level effects of high-potency opioids [67]. This distinction is particularly relevant in fentanyl-dominant environments, where rapid onset and high potency narrow the window for effective reversal.

In addition, macrodosing strategies following naloxone administration may facilitate rapid transition to buprenorphine-based opioid agonist therapy, potentially stabilizing patients after overdose and reducing near-term recurrence risk [68]. Together, these approaches span primary, secondary, and tertiary prevention, forming a coherent pharmacologic continuum.

This framing aligns with harm reduction principles by acknowledging ongoing drug use while seeking to reduce its lethality, rather than conditioning benefit on abstinence. Other opioid antagonists, such as nalmefene, share similar reversal mechanisms but differ in pharmacokinetics and duration of action; however, like naloxone, they function downstream of established respiratory depression and do not modify baseline respiratory risk.

## 6. Clinical and Policy Implications

Viewing buprenorphine through the lens of respiratory risk modification has several implications:

1. Clinical Messaging: Clinicians may more explicitly discuss overdose protection as a benefit of buprenorphine, which may resonate with patients who are ambivalent about abstinence-focused treatment [69].
2. Medication Selection in Ongoing Use: For patients who continue to use illicit opioids, buprenorphine may offer a safety advantage over full agonist therapies (e.g., methadone, slow-release oral morphine) due to its ceiling effect on respiratory depression and partial agonist properties. This supports prioritizing buprenorphine as a first-line agent when overdose risk reduction—rather than opioid abstinence alone—is the primary clinical goal.
3. Low-Threshold Access: Policies that reduce barriers to buprenorphine initiation and continuation—such as same-day starts, flexible dosing models, and reduced administrative burden—may yield population-level mortality benefits beyond traditional treatment metrics [48,70].
4. Guideline Development: Clinical guidelines addressing OIRD and overdose prevention should consider buprenorphine not only as OUD therapy but as part of a broader pharmacologic prevention toolkit [39,60,71,72,73,74,75].
5. Research Priorities: Future studies should explicitly examine respiratory outcomes, overdose severity, and fentanyl-related toxicity in relation to buprenorphine exposure, including partial adherence, intermittent use, and comparative effectiveness versus full agonist therapies.

## 7. Limitations and Cautions

This perspective is intentionally conceptual and should be interpreted within the limits of the available evidence. The central argument—that buprenorphine may function as a pharmacologic modifier of opioid-induced respiratory depression (OIRD)—is grounded in established pharmacology and supported indirectly by observational and experimental data. However, direct causal evidence demonstrating this effect in real-world fentanyl exposure remains limited.

No validated dose threshold has been established at which buprenorphine can be prescribed specifically for the prevention of OIRD, and the present argument should not be interpreted as proposing a guideline-endorsed dosing standard for overdose prevention.

Most population-level evidence cited in support of reduced overdose mortality with opioid agonist therapy is observational and therefore vulnerable to confounding [76]. Individuals receiving buprenorphine differ systematically from those who are not, including in ways that are difficult to measure, such as health-seeking behaviour, stability of drug use, and access to care. As a result, the relative contribution of pharmacologic effects versus behavioural and structural factors cannot be precisely determined.

Importantly, buprenorphine does not eliminate overdose risk. Fatal and non-fatal overdoses continue to occur, particularly in the context of polysubstance use involving benzodiazepines, alcohol, or other sedatives. The protective effects proposed here are therefore probabilistic rather than absolute and should not be interpreted as conferring immunity to respiratory depression.

The mechanistic framework also assumes sufficient receptor occupancy to meaningfully attenuate the effects of high-potency opioids. In practice, variability in adherence, dosing, and pharmacokinetics may limit this effect. Inter-individual differences in opioid sensitivity, including genetic variability in opioid receptor pathways, may further modify both risk of OIRD and response to buprenorphine, representing an important but underexplored area.

Finally, the generalizability of this framework is constrained by context. Much of the supporting evidence arises from North American settings characterized by fentanyl-dominant and polysubstance-contaminated drug supplies. Whether similar dynamics apply in regions with different opioid profiles or patterns of use is uncertain.

Despite these limitations, the absence of definitive causal evidence should not preclude consideration of mechanistically plausible and clinically relevant strategies in a rapidly evolving overdose landscape. The framing proposed here is intended to complement, rather than replace, existing approaches, and to stimulate further empirical investigation into pharmacologic pathways for overdose prevention. 

## 8. Conclusions

In fentanyl-dominant drug markets, opioid-induced respiratory depression represents a pharmacologic problem that may require upstream pharmacologic prevention strategies. Buprenorphine’s partial agonism, ceiling effect, and high receptor affinity position it as a plausible modifier of respiratory risk, not solely a treatment for opioid use disorder. Recognizing this role may strengthen clinical communication, inform medication selection in the context of ongoing opioid use, and support policy approaches aimed at reducing overdose mortality. Further research is needed to quantify these effects and clarify their relative contribution alongside behavioural and structural determinants of risk.

## Data Availability

No new data were created or analyzed in this study. Data sharing does not apply to this article.

## Abbreviations

The following abbreviations are used in this manuscript:

OIRD	Opioid-Induced Respiratory Depression
OUD	Opioid Use Disorder
